# Psychiatric Relapse and Criminal Recidivism of Individuals Found Not Criminally Responsible on Account of Mental Disorder After Absolute Discharge

**DOI:** 10.1177/07067437221116983

**Published:** 2022-08-04

**Authors:** Karine Forget, Pierre Gagné, Schriebert Staco Douyon, Clémence Poirier, Jolène LeBlanc, Marie-Claude Bilodeau, Yann Le Corff

**Affiliations:** 1 Département de psychiatrie, 12370 Université de Sherbrooke, Sherbrooke, Quebec, Canada; 2 Groupe de recherche et d’intervention sur les adaptations sociales de l’enfance de l’Université de Sherbrooke, Sherbrooke, Quebec, Canada; 3 Département de psychiatrie, 90756 Hôpital Pierre Janet, Gatineau, Quebec, Canada; 4 Département de psychiatrie, 157801 Hôpital Sainte-Croix, Drummondville, Quebec, Canada; 5 Département d’orientation professionnelle, Université de Sherbrooke, Sherbrooke, Quebec, Canada

**Keywords:** not criminally responsible, criminal recidivism, psychiatric relapse, absolute discharge

## Introduction

When an accused found not criminally responsible on account of mental disorder (NCRMD) no longer poses a significant threat to the safety of the public as per the Canadian Criminal Code s. 672.54,^1^ an absolute discharge (AD) is granted by the Review Board (RB). Little research, however, is available on the outcomes of these individuals who mostly suffer from a serious mental illness and who will likely require long-term follow-up after AD. Data from the National Trajectory Project (NTP)^2^ showed that the criminal recidivism (CR) rate was relatively low 3 years following the index NCRMD verdict (INCRMDV). Also, the CR rate was twice as high in Quebec (22%) versus British Colombia (10%) and Ontario (9%). The NTP studied CR but not psychiatric relapses (PR) and concluded that information about rehospitalizations was needed considering some new offenses may result in rehospitalization rather than criminal charges. In their one-year follow-up study of absolutely discharged patients in Ontario, Simpson et al.^3^ found a readmission rate of 20% and 5% of CR. More data regarding the outcomes of this population could guide psychiatrists in their recommendations to the RB and assist in the planning of services. The objectives of this study were to examine PR and CR rates of individuals found NCRMD after AD in one region of Quebec, and to identify predictors of PR and CR.

## Methods

In this retrospective longitudinal study, we reviewed the CIUSSSE-CHUS charts (including court-ordered psychiatric evaluations for the INCRMDV and annual reports submitted to the RB), as well as criminal records of 143 individuals found NCRMD in Sherbrooke, Quebec, and who had received an AD between January 1, 2000, and December 31, 2011. The study end date (August 1, 2015) allowed for a minimum of a 3.6-year follow-up (ranging from 3.75 to 15.35 years, excluding 3 people who died less than 3.6 years following AD). Only data from the 3.6-year follow-up were used in the analyses to avoid the bias associated with different observation periods. PR was defined as an emergency room psychiatric consultation or a psychiatric hospitalization occurring after AD. CR was defined as having a conviction or an NCRMD finding for an offence occurring after AD and identified using dockets. Since some individuals assessed in Sherbrooke were followed in their regional hospital, we also reviewed charts from the Granby hospital, the Drummondville hospital, and the Clinique médico-légale de l’Université de Sherbrooke. The study was approved by our institutional ethics board.

## Results

During the 3.6-year follow-up after AD, 25.4% (*n* = 33; 10 MD) of the sample reoffended while 54.3% (*n* = 75; 2 MD) had at least one PR. Overall, 59.7% (*n* = 80; 6 MD) reoffended or had a PR within 3.6 years. Among individuals who had committed an offence against a person as their INCRMDV (*n* = 92), the CR rate was 21.7% (*n* = 20; 5 MD), compared to 30.2% (13/43; 4 MD) for individuals who committed an offence not against a person, but this difference was not statistically significant. Finally, individuals who had PR were more likely to be reoffenders than those who did not (40.0% vs. 6.9%; χ2 (1) = 18.538; *p* < 0.001; ɸ = 0.381). Analyses comparing individuals with and without CR and PR are presented in Table 1.

**Table 1.** Comparison of Individuals With and Without Criminal Recidivism and Psychiatric Relapse.

	Recidivists mean (SD)	Non-recidivists mean (SD)	*t*	*P*	Cohen’s *d*
Age at NCRMD verdict (years)	32.30 (10.14)	35.40 (11.66)	1.361	0.176	0.28
Duration of RB follow-up (days)	329.76 (312.69)	383.06 (425.69)	0.661	0.510	0.14
	*n* (%)	*n* (%)	χ^2^	*P*	ɸ
Sex			0.031	0.860	0.016
Men	27 (81.81%)	78 (80.41%)			
Women	6 (13.04%)	19 (19.59%)			
Index most severe offense			0.797	0.372	0.078
Against a person	20 (60.61%)	67 (69.07%)			
Not against a person	13 (39.39%)	30 (30.93%)			
Diagnosis at the index NCRMD verdict^a^					
Psychotic spectrum disorder	21 (65.63%)	71 (73.20%)	0.674	0.412	0.072
Mood spectrum disorder	11 (34.38%)	21 (21.65%)	2.089	0.148	0.127
SUD	12 (37.50%)	21 (21.65%)	3.176	0.075	0.157
PD	3 (9.38%)	5 (5.15%)	0.737	0.391	0.076
Any Medical follow-up after AD	32 (100.00%)	93 (95.88%)	1.362	0.243	0.103
Psychiatric follow-up after AD	31 (100.00%)	83 (85.57%)	5.024	**0.025**	0.198
Treatment order	10 (31.25%)	16 (16.49%)	3.255	0.071	0.159
Housing order	6 (18.75%)	6 (6.19%)	4.502	**0.034**	0.187
Past criminal convictions or NCRMD findings	25 (78.13%)	56 (60.22%)	3.348	0.067	0.164
	Psychiatric relapse mean (SD)	No psychiatric relapse mean (SD)	*T*	*P*	Cohen’s *d*
Age at NCRMD verdict (years)	33.29 (10.97)	37.51 (12.12)	2.143	**0.034**	0.37
Duration of RB follow-up (days)	384.07 (371.46)	354.35 (424.60)	0.438	0.662	0.07
	n (%)	n (%)	χ^2^	*P*	ɸ
Sex			0.003	0.955	0.005
Men	61 (81.33%)	51 (80.95%)			
Women	14 (18.67%)	12 (19.05%)			
Index most severe offense			0.339	0.560	0.050
Against a person	47 (63.51%)	43 (68.25%)			
Not against a person	27 (36.49%)	20 (31.75%)			
Diagnosis at the index NCRMD verdict^a^					
Psychotic spectrum disorder	50 (68.49%)	46 (73.02%)	0.333	0.564	0.049
Mood spectrum disorder	20 (27.40%)	15 (23.81%)	0.228	0.633	0.041
SUD	22 (30.14%)	12 (19.05%)	2.218	0.136	0.128
PD	6 (8.22%)	2 (3.17%)	1.554	0.212	0.107
Any Medical follow-up after AD	73 (97.33%)	60 (96.77%)	0.037	0.847	0.017
Psychiatric follow-up after AD	71 (95.95%)	50 (81.97%)	7.029	**0.008**	0.228
Treatment order	22 (29.33%)	7 (11.11%)	6.850	**0.009**	0.223
Housing order	11 (14.67%)	1 (1.59%)	7.377	**0.007**	0.231
Past criminal convictions or NCRMD findings	45 (61.64%)	37 (63.79%)	0.064	0.801	0.022

*Note*. Total sample size = 140; AD = absolute discharge; NCRMD = not criminally responsible on account of mental disorder; PD = personality disorder; ɸ = phi coefficient; RB = review board; SD = standard deviation; SUD = substance use disorder; *t* = *t* statistic for independent-sample *t*-test; χ^2^ = chi-square statistic.

^a^ The two most important diagnoses at the time of the index NCRMD were noted, thus a subject can count in more than one diagnostic category.

## Discussion

We found a similar CR rate at 3.6 years following AD compared to 3 years following the INCRMDV in Quebec (NTP^2^), which is reassuring because one could have hypothesized that CR would increase without RB supervision. Our 3.6-year CR and PR rates were, respectively, 5 times higher and 2.7 times higher than the 1-year rates observed in Ontario,^3^ which could be explained by our longer follow-up (CR may be lower in the first year following AD but could increase afterwards), missing data in our study (see limitations below) or a broader use of NCRMD verdict in Quebec leading to the inclusion of patients with potentially less severe diseases. Personality and substance use disorders at the INCRMDV were not associated with CR or PR, which differs from other studies,^3,4^ and could be explained by the low numbers of both diagnoses in our sample. The presence of psychiatric follow-up and housing order was associated with both CR and PR, potentially indicating a more disabling illness. Finally, our study showed that PR was correlated to CR. These results support the importance of maintaining adequate follow-up and tailored services after AD.

Documenting rehospitalization was a strength of this study considering some offenses may not result in criminal charges but rather in hospital visits. However, rehospitalization might have happened before an offence was committed, thus demonstrating good risk management through the prevention of the offence. Our small sample could have restrained statistical power. We had however a longer follow-up compared to similar studies, and we considered the impact of medical follow-up and treatment/housing orders on recidivism. Other limitations are the potential missing information from files and our recidivism measure that may not capture all new offenses. Finally, clinical outcomes were confined to clinical contacts in hospitals in our immediate vicinity, leaving out other potential contacts throughout Quebec and those in prison.

## Abbreviations

AD: Absolute discharge
CIUSSSE-CHUS: Centre intégré universitaire de santé et de services sociaux de l’Estrie – Centre hospitalier universitaire de Sherbrooke
CR: Criminal recidivism
INCRMDV: Index not criminally responsible on account of a mental disorder verdict
MD: Missing data
NCRMD: Not criminally responsible on account of a mental disorder
NTP: National Trajectory Project
PR: Psychiatric relapse
RB: Review Board
